# Matching *ex vivo* MRI With Iron Histology: Pearls and Pitfalls

**DOI:** 10.3389/fnana.2019.00068

**Published:** 2019-07-03

**Authors:** Amaury De Barros, Germain Arribarat, Jeanne Combis, Patrick Chaynes, Patrice Péran

**Affiliations:** ^1^Toulouse NeuroImaging Center, University of Toulouse Paul Sabatier-INSERM, Toulouse, France; ^2^Department of Anatomy, Toulouse Faculty of Medicine, Toulouse, France

**Keywords:** iron quantification, *ex vivo*, magnetic resonance imaging, iron histology, QSM

## Abstract

Iron levels in the brain can be estimated using newly developed specific magnetic resonance imaging (MRI) sequences. This technique has several applications, especially in neurodegenerative disorders like Alzheimer's disease or Parkinson's disease. Coupling *ex vivo* MRI with histology allows neuroscientists to better understand what they see in the images. Iron is one of the most extensively studied elements, both by MRI and using histological or physical techniques. Researchers were initially only able to make visual comparisons between MRI images and different types of iron staining, but the emergence of specific MRI sequences like R2^*^ or quantitative susceptibility mapping meant that quantification became possible, requiring correlations with physical techniques. Today, with advances in MRI and image post-processing, it is possible to look for MRI/histology correlations by matching the two sorts of images. For the result to be acceptable, the choice of methodology is crucial, as there are hidden pitfalls every step of the way. In order to review the advantages and limitations of *ex vivo* MRI correlation with iron-based histology, we reviewed all the relevant articles dealing with the topic in humans. We provide separate assessments of qualitative and quantitative studies, and after summarizing the significant results, we emphasize all the pitfalls that may be encountered.

## Introduction

Iron is an essential cofactor in many physiological processes. In the brain, it contributes to myelination, DNA synthesis, mitochondrial respiration, oxygen transport, and neurotransmitter metabolism (Ward et al., 2014). The iron redox couple Fe^2+^/Fe^3+^ mediates many reactions, the precise redox potential varying according to the nature of the ligand. Fe^2+^ is essentially present in the labile iron pool in the cytosol, which represents the iron that is directly available for cellular chemistry. Fe^3+^, which is insoluble, is the major form of iron in the brain, and is mainly found in iron-binding proteins. Hemoproteins (hemoglobin, myoglobin, cytochromes) represent a large proportion of iron in the whole body, but only a minor proportion of iron in the brain parenchyma, except in large vessels. Non-heme iron proteins can be classified according to their function. Transferrin (TF) is the main protein involved in iron uptake by the transferrin-transferrin receptor pathway. TF has two Fe^3+^ binding sites, but in physiological conditions, only 30% of TF molecules are loaded (Hare et al., 2013). Transferrin receptor (TfR 1 and 2) are expressed in all the central nervous system and especially in the plasma membrane of the neurons. The non-transferrin bound iron pathway is a minority pathway including several channel proteins and ligand/receptors interactions, notably Divalent Metal Transporter 1 (a channel protein) responsible for ferrous iron uptake. Ferroportin (FP) is the only known iron export protein. FP exports Fe^2+^ from the cytosol, and Fe^2+^ is further oxidized by hephaestin or ceruloplasmin and their ferroxidase activity. Both are expressed in the brain (Jiang et al., 2017). Hepcidin regulates systemic iron homeostasis by direct interaction with FP. In the cytosol, except in the labile iron pool, iron is ligated by low molecular weight proteins such citrate or ascorbate, iron-sulfur proteins, or ribonucleotides. Ferritin is the major storage protein. It is composed of two subunits, heavy (H) and light (L) chain, that surround a ferrihydrite core capable of storing up to 4,500 iron atoms. In physiological conditions, however, only 2,000–2,500 atoms are usually stored (Bossoni et al., 2017). It should be noted that ferritin may also be iron-free, especially in a pro-inflammatory condition. In the brain, oligodendrocytes (cells producing myelin) are the cells with the most intense immunostaining for ferritin in normal conditions because of their high iron metabolism for axonal myelination. They express both L and H subunits, like microglial cells. The neurons essentially express H subunits. The composition of ferritin in astrocytic cells has yet to be determined (Jiang et al., 2017). L-subunit is required for the long-term storage of iron, whereas H-subunit has ferroxidase activity. The H/L ratio increases with age by a factor 2–3 (Connor et al., 1995).

Magnetite was recently discovered in the brain, and may substitute the ferrihydrite crystal in ferritin in pathological conditions such as Alzheimer's disease (AD) (Bossoni et al., 2017), although this is quite controversial, as some authors claim that the magnetite crystal is too large (Kumar et al., 2016). Magnetite has a magnetic moment 10 times greater than that of ferrihydrite, but seems to be a thousand times less concentrated (Bulk et al., 2018b). Despite its rather misleading name, hemosiderin contains non-heme iron (Harrison and Arosio, 1996). Hemosiderin is regarded as a ferritin degradation product, as immunolabeling studies have found ferritin expressed in siderosomes. Siderosomes are structures that appear to be derived from lysosomes that have degraded ferritin to form hemosiderin in excess iron conditions. In the brain, hemosiderin can be also found during hemorrhages (hematomas, microbleeds, etc.) or in pathological conditions involving excess iron (Harrison and Arosio, 1996; Quintana et al., 2006). Iron is mainly present in the form of ferrihydrite crystals, as it is in ferritin. Hemosiderin is regarded as insoluble, unlike ferritin, and has a high iron/protein ratio. The protein part is less clearly structured than that of ferritin.

Neuromelanin and lipofuscin are two lysosomal products. Neuromelanin is a black, insoluble pigment incorporated into 0.5–3 μm intracytoplasmic organelles. These organelles are composed of proteins, lipids and neuromelanin granules of 200–400 nm with 30-nm substructures. Although the composition and appearance of neuromelanin vary in electron microscopy, depending on the brain region from which it is extracted, the 30-nm substructure is remarkably well-preserved (Zecca et al., 2008; Bush et al., 2009; Engelen et al., 2012). It comprises a eumelanin envelope with a low oxidative potential surrounding a pheomelanin core, with a pheomelanin/eumelanin ratio of 3/1 (Zecca et al., 2008). Neuromelanin is now known to be produced by catecholaminergic neurons in response to excessive cytoplasmic accumulation of unsupported catecholamines in synaptic vesicles. Aside from its role in catecholamines catabolism, neuromelanin is a chelator of heavy metals and environmental toxins (Zucca et al., 2017). In terms of absolute concentration, the most chelated metal by far (by a factor of at least 10^3^ relative to the others) is Fe^3+^. However, the number of chelated metals is extremely varied. It is in the melanin-containing neurons of the susbtantia nigra that we find the most neuromelanin, with a higher iron concentration than elsewhere. These neurons contain very little ferritin, compared with non-melanin-containing neurons and adjacent glia (Zecca et al., 2004). Neuromelanin has two iron binding sites: a high-affinity site for storing iron in an inactive redox form, which thus participates in its antioxidant action; but also a lower-affinity site that comes into play when the cell is in an iron overload situation. This site is responsible for the easier release of iron and thus contributes to the pro-oxidative properties of neuromelanin. It is now generally accepted that neuromelanin has a dual action (Faucheux et al., 2003; Zucca et al., 2017). There has recently been renewed interest in this substance, partly because of its involvement in the pathophysiology of Parkinson's disease (PD) and related pathologies, and partly because modern imaging techniques allow it to be detected and quantified *in vivo*, and can thus yield evidence of its variations as a function of time and pathology. This is the case in magnetic resonance imaging (MRI) with fast spin echo techniques involving magnetization transfer (Sasaki et al., 2006, 2008) as well as in positron emission tomography (PET) (Hansen et al., 2016). The iron concentration of neuromelanin has also been experimentally shown to be correlated with T1 and T2 relaxation (Trujillo et al., 2016).

Lipofuscin is another insoluble and ubiquitous pigment (yellow-brown) of the brain. Lipofuscin differs from neuromelanin in its autofluorescence with ultraviolet light (Double et al., 2008). It is also the result of autophagocytosis by the lysosomal system, including autophagocytosis of mitochondria. The final organelle therefore has a size comparable to that of neuromelanin. It is rather perinuclear compared with neuromelanin, which can be found at axonal endings. Lipofuscin increases with age and is a marker of neuronal aging. It is also involved in the chelation of metals such as iron. The presence of free iron contributes to an increase in lipofuscin, probably through lipid peroxidation (Ashraf et al., 2018). Although they would appear to be quite similar, in terms of their formation mechanism and their structure (proteins, lipids, metal ions) lipofuscin and neuromelanin actually seem quite distinct. Moreover, there is no lipofuscin in neurons containing neuromelanin (Terman and Brunk, 2004; Double et al., 2008; Sulzer et al., 2008). There are no scientific data on the potential role of lipofuscin in relaxation in MRI and there are no specific designed sequences for lipofuscin compare to neuromelanin, but we can assume that it does indeed play a role, by virtue of its nature and its proximity to neuromelanin.

In physiological conditions, iron homeostasis is strictly regulated, but in many pathological processes, it is dysregulated, leading to the formation of reactive oxygen species implicated in oxidative stress. The main pathologies concerned are extrapyramidal disorders such as PD or multiple system atrophy (MSA), AD, multiple sclerosis (MS), and specific iron metabolism disorders grouped under the heading of *neurodegeneration with brain iron accumulation* (Dusek et al., 2012). Aging is the main factor favoring accumulation of iron in the brain. Iron increases during the first decades, reaching maximum values during the fifth or sixth decade with only subtle changes after (HALLGREN and SOURANDER, 1958).

## Iron Detection Using Histological or Physical Techniques

Ever since the pioneering work carried out around the turn of the Twentieth-century by Zaleski (1887) and Spatz (1922) (Koeppen, 1995), brain iron quantification and characterization has been an important topic of research. Histological methods such as Perls' or Turnbull staining are the best known techniques. Perls' stain reacts mainly with Fe^3+^ ions, giving an intense Prussian (or Berlin) blue. However, it also reacts with Fe^2+^, resulting in a white precipitate known as Everitt's salt that slowly oxidizes to give Prussian blue (Meguro et al., 2007). Perls' staining only makes it possible to highlight non-heme iron. The iron present in the heme has strong covalent bonds and only treatment with strong alkalis (ammonium sulfide) can dissociate it (Meguro et al., 2007). By contrast, the iron bound to iron metabolism molecules (ferritin, hemosiderin, transferrin, iron-sulfur proteins, etc.) can easily be ionized from a pH of around 4.5. This explains why treatment with hydrochloric acid is necessary for Perls' staining. Like iron heme, iron present in neuromelanin and probably lipofuscin is historically described as *invisible* iron, owing to its strong affinities. As a result, it cannot be revealed by Perls' staining. The second best known histochemical method is the Turnbull technique, which consists in applying potassium ferricyanide instead of potassium ferrocyanide, again in association with hydrochloric acid. This treatment makes it possible to reveal Fe^2+^ ions. Other methods involve using the iron-sulfur reaction to demonstrate both Fe^2+^ and Fe^3+^. Treatment with ammonium sulfide reveals Fe^2+^ (Quincke method), and pretreatment with ammonium sulfide and secondary treatment with Turnbull stain reveal Fe^3+^ in addition to Fe^2+^ (Tirmann-Schmelzer method). The Tirmann-Schmelzer method is an iron-sulfur method. Theoretically, the iron-sulfur technique has three drawbacks. First, not all Fe^3+^ is converted to Fe^2+^, thus limiting the sensitivity of the technique. Second, ammonium sulfide treatment also extracts heme iron in theory but only if the treatment is at particular low pH and/or for a very long period (several days) compare to classical protocols (90 min). Third, sulfur-based methods are not specific for iron and causes the precipitation of other metal ions, such as copper or zinc into copper sulfide or zinc sulfide. However, these precipitates do not further react with potassium ferricyanide (Turnbull reaction). So, while the simple iron-sulfur techniques react with zinc and copper, the actual Tirmann-Schmelzer method does not suffer from this lack of specificity, because the specificity for iron comes from the reaction of Fe^2+^ ions with potassium ferricyanide (Meguro et al., 2007). The Tirmann-Schmelzer method has recently been used to study iron in rigorous studies with good results (Wiggermann et al., 2017; Hametner et al., 2018).

**Figure 1:**
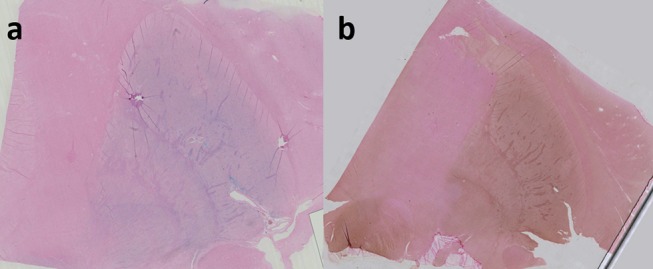
Perls' staining of right lenticular nucleus in coronal plane **(a)**; Perls + DAB staining of right lenticular nucleus **(b)**. both Perls and Perls + DAB were counterstained with nuclear fast red. Image obtained during experimental procedures with all appropriate ethical approval. Included for illustrative purposes.

To enhance the visualization (augmented sensitivity) of Perls' or Turnbull staining, diaminobenzidine (DAB) can be added, turning Perls' or Turnbull's blue to a brown palette (cf. Figure 1). Another colorimetric assay method involves reacting ferrozine with Fe^2+^ to give a magenta red precipitate that can then be assayed spectrophotometrically. Pretreatment with hydroxylamine hydrochloride converts Fe^3+^ to Fe^2+^.

Although the iron itself cannot be revealed by immunohistochemistry, the same is not true of the proteins containing this iron. The advantage of this technique is that it identifies antigens (generally proteins) of interest at the cellular or extracellular level using specific antibodies. These antibodies may be of either polyclonal or monoclonal origin. Polyclonal antibodies recognize various antigens of the protein of interest. There is therefore a risk of cross-reactions. Monoclonal antibodies recognize a single antigen. They have greater specificity, but diminished sensitivity.

The main proteins that can be targeted are transferrin, FP, iron-responsive proteins, and ferritin. There are both polyclonal and monoclonal antibodies for ferritin. However, each antibody is specific for either the L chain, the H chain, or mitochondrial ferritin.

In conclusion, there are no perfect staining methods for accurately calculating the iron concentration of a given tissue. They are therefore considered to be semiquantitative methods at best. Their strength lies in their qualitative character to highlight areas rich in iron with an excellent spatial resolution, whether macroscopically or microscopically.

Physical techniques like inductively coupled plasma mass spectrometry (ICP-MS) or atomic absorption spectroscopy (AAS) are used to quantify iron concentrations with precision, but they cannot map the iron in the brain. Recent techniques such as laser ablation inductively coupled plasma mass spectrometry (LA-ICP-MS) or X-ray fluorescence (XRF) can robustly quantify *and* precisely map iron in brain structures (Matusch et al., 2011; Zheng et al., 2013).

## MR Signal and Iron-Binding Proteins

Ferritin, hemosiderin and deoxyhemoglobin are considered to be the main forms of iron present in sufficient quantities to affect MR contrast in the human brain (Schenck, 2003; Haacke et al., 2005). To a lesser extent, neuromelanin can also affect the MRI signal in particular locations. Lipofuscin's signal impact needs more investigation. Proteins associated with small numbers of iron atoms like transferrin are often regarded as negligible. Consider the case of ferritin. It is not so much its total composition that is important as its core, most often a ferrihydrite crystal which, like all metallic nanoparticles, has magnetic properties capable of affecting susceptibility. Ferrihydrite is an antiferromagnetic structure with, by definition, antiparallel magnetic ordering with moment compensation. The magnetization vector in a crystal is not isotropic, as the direction of spin relative to the crystal lattice depends on magnetocrystalline anisotropy. This anisotropy stems from the presence of *easy magnetization* directions within the crystal. However, the nanometric size of the core (about 6 nm) gives it different properties. Below a certain size, particle magnetization relaxes above the energy barriers of anisotropy. If the volume of the particle is small enough, the energy barriers separating the minimum energy directions (easy directions) may correspond to thermal energy at room or body temperature. The magnetic moment can fluctuate from one easy direction to another. This thermally activated phenomenon constitutes superparamagnetic relaxation (Daou, 2007; Bennett et al., 2008).

It should also be noted that the particle's magnetic moment depends on the number of iron atoms and surface/volume ratio in ferritin. The maximum capacity is 4,500 atoms, but the actual capacity often oscillates around 2,000 atoms. Finally, the crystal is not always in the form of ferrihydrite, as it can also be in the form of magnetite/maghemite, which has a magnetic moment 10 times greater than the crystal of ferrihydrite.

Magnetic susceptibility, denoted as chi (χ), is defined as the ratio of the magnetic moment (M) to the magnitude of the external magnetic field (B). This variable is dimensionless.

$$\begin{array}{l} \chi \;=\;M\;/\;B \end{array}$$

Its molar magnetic susceptibility (χm) is equal to its magnetic susceptibility (χ) multiplied by the molar mass of the test compound.

Diamagnetic substances have a molar susceptibility of <1 ppm. This is the case of most human tissue, which is very weakly diamagnetic. By contrast, paramagnetic and superparamagnetic substances have a molar susceptibility >1 ppm, and some substances, such as ferritin, have a very high molar susceptibility (5,000 ppm).

In other words, highly (super- or ferro-) paramagnetic substances significantly increase the magnetic field at the local level. This increase in the magnetic field results in changes in the frequency of spin precession, and thus in a poor location of the MR signal (image distortion). These local inhomogeneities of the magnetic field also have the effect of shortening the T2^*^ or T2 times, by accelerating the spin phase shifts in between.

This is called the *susceptibility artifact*. Magnetic susceptibility, phase shift and shortening of T2 ^*^ are the main approaches to the quantification of iron in MRI.

## Iron Estimation by MRI

Advances in MRI mean that we are now able to estimate the presence of iron in the brain. Several techniques have emerged to quantify iron content. Some are based on transverse relaxation time (T2, T2^*^ and T2') or its inverse (R2, R2^*^ and R2'), in the relation R2^*^ = R2 + R2'. Iron, especially when it is stored in ferritin or hemosiderin, induces local magnetic field inhomogeneities and increases R2 and R2^*^ relaxation rates. Transverse relaxation rates (R2 or R2^*^) can be measured by multi-echo acquisition (spin echo for R2 and gradient echo for R2^*^), then voxelwise fitting to an exponential model (Péran et al., 2007).

The second type of sequences used to estimate iron are based on paramagnetic effects from iron-containing proteins that increase bulk tissue magnetic susceptibility. Magnetic susceptibility information is contained in phase images. Susceptibility weighted imaging (SWI), which combines phase images with magnitude images to enhance contrast among structures with different local susceptibilities, was the first to be used. Quantitative susceptibility mapping (QSM) was developed more recently to directly measure susceptibility and be independent of imaging parameters such as echo time or field strength (de Rochefort, 2010; Deistung et al., 2016). QSM is based on phase images and uses complex post-treatment algorithms to remove background field components after phase unwrapping. Several algorithms and methods can be used, such as Laplacian-based methods, calculation of susceptibility using multiple orientation sampling (COSMOS), morphology-enabled dipole inversion (MEDI), and sophisticated harmonic artifact reduction on phase data (SHARP) method (Wang and Liu, 2015; Deistung et al., 2016). QSM allows investigators to distinguish between calcium and iron, unlike R2^*^. There are other sequences, such as field-dependent R2 increase (FDRI), that have high sensitivity and specificity for iron, but they are seldom used in clinical practice, owing to technical constraints.

Iron-based sequences are used in research or clinical routine in healthy participants (Péran et al., 2009) to estimate the amount of iron in brain structures, especially the basal ganglia, or to compare patients with diseases such as PD with healthy controls (Péran et al., 2010) or establish differential diagnoses for disorders such as MSA (Barbagallo et al., 2016; Péran et al., 2018).

## Iron–MRI Correlation

Researchers trying to correlate iron-based sequences with an estimation of iron concentration in brain structures draw on data from a seminal work published in 1958 by HALLGREN and SOURANDER (1958) and verified in recent research (Krebs et al., 2014), using regression line equations for many parts of the brain, where y = concentration of non-heme iron per 100 g fresh weight and x = age of participant. For all types of iron-based sequences [FDRI Bartzokis et al., 2007,R2^*^Péran et al., 2009, or QSM Bilgic et al., 2012], correlations are calculated with Hallgren and Sourander' s equations.

Despite these rigorous data, the desire to correlate estimations of iron with MRI using a reference quantitative technique has led scientists to undertake *ex vivo* MRI, which allows for further investigations after scanning. *Ex vivo* MRI has many advantages compared with *in vivo* MRI, including longer acquisition time for better spatial resolution, air-tissue interface elimination, no importance of the specific absorption rate (SAR), no patient artifacts, and the possibility of using a dedicated receiver coil for small anatomical specimens. *Ex vivo* MRI has gained in popularity in the scientific community, not only for anatomical atlases and forensic medicine, but also for molecular and trace element mapping.

The aim of the present literature review was to systematically describe and evaluate published studies that used *ex vivo* MRI combined with histological or physical techniques to detect iron in the brain. We focused on quantitative studies that attempted to find statistical correlations between the two kinds of techniques, but also included articles exploring iron in relation to a specific pathology or just for anatomical description purposes. After summarizing their results, we discuss all the pitfalls that can be encountered along the way.

## Methods

**Figure 2:**
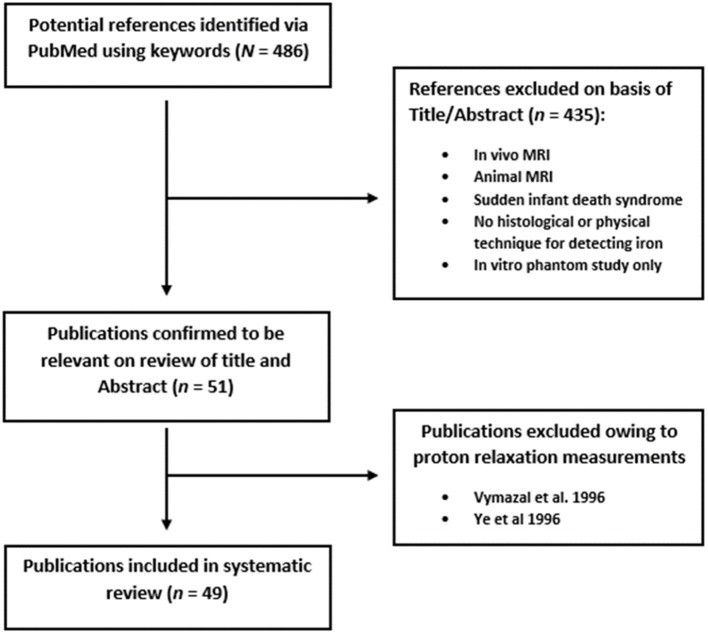
Flowchart depicting systematic review process.

The initial search was undertaken on PubMed using the following terms: “brain” AND “magnetic resonance imaging” OR “MRI” OR “MR” OR “QSM” OR “R2^*^” AND “*Ex-vivo*” OR “post-mortem” OR “postmortem” AND “iron.” The review was carried out in accordance with PRISMA guidelines for systematic reviews (Moher et al., 2009). Our search criteria yielded 486 results. We limited the articles we reviewed to human studies and papers written in English. Relevant papers were identified by manual review of titles and abstracts. Articles with no evidence of iron staining or detection by a physical technique on the MR scanned specimen were excluded. A total of 51 articles were fully analyzed, and two were excluded owing to the use of proton relaxation measurements rather than MRI (Vymazal et al., 1996; Ye et al., 1996). We distinguished quantitative analyses from qualitative (visual assessment) ones in the 49 final articles and listed all the different pathologies concerned (Figure 2). Relevant data were listed. In the case of the quantitative studies, we identified the linear regression parameters, along with their linear equation parameters. Finally, key methodological points were noted, in order to highlight limitations that may be encountered when matching *ex vivo* MRI with histology.

## Results

Table 1 sums up all the recent literature on post mortem MRI and histological or physical techniques for measuring iron. Papers were classified as quantitative (25 articles) when correlations were sought between MR data and histological or physical techniques, and qualitative (24 articles) when images were simply visually assessed. Where articles described two reference techniques (often a histological one and a physical one), the quantitative assessment was only applied to the physical technique. In some papers, quantitative measures were carried out in order to compare data between the pathological group and controls, so they were not classified as quantitative.

**Table 1:** List of all references featuring iron descriptions by both MRI and histological/physical technique.

**References**	**Iron sequences / field strength (tesla)**	**Histology or physical technique**	**Brain structure** **(fresh, frozen or fixed when imaged)**	**Control or pathology (type)**	**Sample size**	**Qualitative or quantitative**
Chen et al., 1989	T2 (1.5T) (MRI + NMR)	Iron: Perls and AAS Ferritin: peroxidase-antiperoxidase method and radio-immune assay kit	Fresh brains	Control	10	Qualitative and quantitative
Dhenain et al., 2002	T2^*^ (11.7T) (MRI + NMR)	Iron: Perls + DAB	Superior temporal gyrus / Senile plaques (fixed)	AD Control	2 2	Qualitative
Fujii et al., 2007	T2 (3T)	Iron: PerlsFerritin: IHC	Putamen (fixed)	Control	4	Qualitative
House et al., 2007	R2 (4.7T)	Iron: AAS	10-mm coronal slices of thawed hemispheres	AD Control	4 2	Quantitative
House et al., 2008	R1 (1.4T) R2 (1.4T) (MRI+ NMR)	Iron: Perls and ICP-AES	10-mm coronal slices of thawed hemispheres	AD Possible Alzheimer Control	3 2 5	Quantitative (ICP-AES) Qualitative (Perls)
Matsusue et al., 2008	T2 (1.5, 3T)	Iron: PerlsFerritin: IHC	Putamen (fixed)	MSA	7	Qualitative
Tatsumi et al., 2008	T2 (1.5T) T2^*^ (1.5T)	Iron: Perls	Brain (fixed)	Microbleeds	1	Qualitative
van Rooden et al., 2009	T2^*^ (7T)	Iron: Perls + DAB	10-mm coronal slices of brains (fixed)	AD CAA Control	6 7 5	Qualitative
Matsusue et al., 2009	T2 (1.5T)	Ferritin: IHC	Cerebellum (fixed)	MSA	7	Qualitative
Yao et al., 2009	R2^*^ (1.5,3,7T)	Iron: ICP-MS	Brains (fixed)	Control	2	Quantitative
Fukunaga et al., 2010	R2^*^ (7T) Phase (7T)	Iron: Perls + DABFerritin: IHC	Parietal cortex (fixed) Occipital cortex (fixed)	Control	1	Qualitative
Hopp et al., 2010	SWI (1.5T) Phase (1.5T)	Iron: XRF	Fixed brains	PD AD TBI PSP PMA	1 1 1 1 1	Qualitative (SWI) and quantitative (Phase)
Langkammer et al., 2010	R2 (3T) R2^*^ (3T)	Iron: ICP-MS	Fresh brains	Control	7	Quantitative
Schrag et al., 2010b	SWI (3T)	Iron: Perls + DAB	10-mm coronal slices of brains/Microbleeds (fixed)	AD + CAA AD CAA Control	4 2 2 2	Qualitative
Bagnato et al., 2011	R2^*^ (7T) Phase (7T)	Iron: Perls + DAB; Turnbull + DAB + Ammonium sulfideFerritin: IHC (polyclonal, light chain)	10-mm coronal slices (fixed)	MS Control	2 1	Qualitative
De Reuck et al., 2011	T2 (7T) T2^*^ (7T)	Iron: Perls	Whole brains (fixed)	AD with cerebral bleeds	20	Qualitative
Antharam et al., 2012	R2 (14.1T) R2^*^ (14.1T)	Iron: XRF	Hippocampus (frozen)	AD Control	5 3	Quantitative
Kwan et al., 2012	R2^*^ (7T)	Iron: Perls + DABFerritin: IHC	Motor cortex (fixed)	ALS	1	Qualitative
Langkammer et al., 2012	QSM (3T)	Iron: ICP-MS	Whole brains (fresh)	Control	13	Quantitative
Langkammer, 2012	R2 (3T) R2^*^ (3T) Phase (3T)	Iron: ICP-MS	Frontal lobe (fresh) Occipital lobe (fresh)	Control	6	Quantitative
Liem et al., 2012	T2 (7T) T2^*^ (7T)	Iron: Perls	10-mm coronal brain slices (fixed)	CADASIL	3	Qualitative
Massey et al., 2012	T2 (9.4T)	Iron: Perls	STN (fixed)	Control	8	Qualitative
Zheng et al., 2012	T2^*^ (3T) Flair (3T) SWI (3T)	Iron: XRF	10-mm coronal brain slices (fixed)	Stroke	3	Qualitative
Blazejewska et al., 2013	T2^*^ (7T)	Iron: Perls	Substantia nigra (fixed)	Parkinson Control	1 2	Qualitative
Foroutan et al., 2013	T2 (21.1T) T2^*^ (21.1T)	Iron: PerlsFerritin: IHC (light chain)	Basal ganglia and midbrain (fixed)	PSP Control	6 3	Qualitative
Walsh et al., 2013	R2 (4.7T) R2^*^ (4.7T) Phase (4.7T)	Iron: Perls	Basal ganglia and MS lesions (fresh)	MS	3	Quantitative
Zheng et al., 2013	QSM (3T)	Iron: XRF	5-mm coronal brain slice (fixed)	MS	1	Quantitative
Meadowcroft et al., 2015	T2^*^ (7T) R2^*^ (7T)	Iron: Perls + DAB	60-μm entorinal cortex slices (fixed)	AD Control	5 3	Qualitative
Stüber et al., 2014	R1 (7T) R2^*^ (7T) QSM (7T)	Iron: Perls and PIXE	Occipital lobe (fixed) Paracentral cortex (fixed) STN (fixed)	Control	3	Quantitative (PIXE) Qualitative (Perls)
Tan et al., 2014	QSM (3T)	Iron: ICP-MS	Cavernomas (fixed)	Cavernomas	4	Quantitative
Birkl et al., 2015	R2' (3T) TCR2' (3T)	Iron: SQUID magnetometry and ICP-MS	Whole brain (fresh)	Control	5	Quantitative
Sun et al., 2015	QSM (4.7T)	Iron: Perls	Whole brain (fresh)	Control	2	Quantitative
Wisnieff et al., 2015	R2^*^ (3T) QSM (3T)	Iron: Perls and LA-ICP-MS	MS lesions (fixed)	MS	1	Quantitative (LA-CP-MS) Qualitative (Perls)
Dusek et al., 2016	T2^*^ (7T) R2^*^ (7T)	Iron: Turnbull + DAB + Ammonium sulfide and AAS Ferritin: IHC	Basal ganglia (fixed)	WilsonControl	9 6	Quantitative Qualitative (Ferritin)
Lee et al., 2016	T2^*^ (7T)	Iron: Perls	Substantia nigra (fixed)	Control	2	Quantitative
van Veluw et al., 2016	T2 (7T) T2^*^ (7T)	Iron: Perls	5-10-mm coronal brain slices / Microbleeds (fixed)	CAA	5	Qualitative
Wallace et al., 2016	R2^*^ PSIR	Iron: Perls + DAB, Turnbull + DAB	Whole brain / Occipital lobe / Transverse temporal gyrus (fixed)	Control	2	Quantitative
Birkl et al., 2017	R2^*^ (3T) TcR2^*^ (3T)	Ferritin: IHC (light chain)	10-mm coronal brain slices (fixed)	MS Control	3 1	Quantitative
Dal-Bianco et al., 2017	T2 (7T) Phase (7T) SWI (7T)	Iron: Turnbull + DAB	MS lesions (fixed)	MS	4	Qualitative
De Reuck et al., 2017	T2^*^ (7T)	Iron: Perls	Coronal brain slices (fixed)	ALS FTLD Control	12 38 28	Qualitative
Massey et al., 2017	T2 (9.4T)	Iron: Perls	Substantia nigra (fixed)	Control PD PSP	10 5 8	Qualitative
Matsuda et al., 2017	T1 (7T) T2 (7T) T2^*^ (7T)	Iron: Perls	Ganglioglioma (fixed)	Ganglioglioma	1	Qualitative
Wiggermann et al., 2017	QSM (3T)	Iron: Turnbull + ammonium sulfide + DAB	MS lesions (fixed)	MS	5	Quantitative
Bagnato et al., 2018	R2^*^ (7T)	Iron: Turnbull + DAB + Ammonium sulfide	10-mm coronal brain slices (fixed)	MS	7	Quantitative
Bulk et al., 2018b	R2^*^ (7T)	Iron: EPR and SQUID	Medium temporal gyrus (fixed)	AD Control	1 1	Quantitative
Bulk et al., 2018a	T2^*^ (7T)	Iron: Perls + DAB	Medium frontal gyrus (fixed)	AD Control	21 13	Quantitative
Hametner et al., 2018	R2^*^ (7T) QSM (7T)	Iron: Turnbull + ammonium sulfide + DAB and ferrozine assay	Whole brain (fresh)	Control	6	Quantitative
Kaunzner et al., 2019	QSM (3T)	Iron: Perls + DAB	MS lesions	MS	7	Qualitative
Lee et al., 2018	T1map T2map T2^*^map T2^*^/T2 T2^*^/T2^2^ SWI QSM	Iron: Perls	Substantia nigra (fixed)	Control	5	Quantitative

AAS, atomic absorption spectrometry; AD, Alzheimer's disease; ALS, amyotrophic lateral sclerosis; CAA, cerebral amyloid angiopathy; DAB, diaminobenzidine; EPR, electron paramagnetic resonance; FTLD, frontotemporal lobar degeneration; ICP-AES, inductively coupled plasma atomic emission spectrometry; ICP-MS, inductively coupled plasma mass spectrometry; IHC, immunohistochemistry; LA-ICP-MS, laser ablation ICP-MS; MSA, multiple system atrophy; MS, multiple sclerosis; NA, not available; PD, Parkinson's disease; PIXE, particle induced X-ray emission; PMA, progressive muscular atrophy; PSIR, T1 phase sensitive inversion recovery; PSP, progressive supranuclear palsy; QSM, quantitative susceptibility mapping; SQUID, superconducting quantum interference device magnetometer; STN, subthalamic nucleus; SWI, susceptibility weighted images; TBI, traumatic brain injury; TcR2^*^, temperature coefficient R2^*^; XRF, X-ray fluorescence.

MR scanners varied widely in main field strength (1.4–21.1 tesla), with the largest, supported by micro-MRI, being particularly useful for small specimens. Many MRI techniques were used to highlight iron, but two recent techniques (R2^*^ and QSM) seemed particularly valuable. A total of 17 articles published after 2009 featured R2^*^ techniques (Yao et al., 2009; Fukunaga et al., 2010; Langkammer et al., 2010; Bagnato et al., 2011, 2018; Antharam et al., 2012; Kwan et al., 2012; Langkammer, 2012; Walsh et al., 2013; Stüber et al., 2014; Dusek et al., 2015; Meadowcroft et al., 2015; Wisnieff et al., 2015; Birkl et al., 2017; Bulk et al., 2018b; Hametner et al., 2018), while 10 published after 2012 used QSM techniques (Langkammer et al., 2012; Zheng et al., 2013; Stüber et al., 2014; Tan et al., 2014; Sun et al., 2015; Wisnieff et al., 2015; Wiggermann et al., 2017; Hametner et al., 2018; Lee et al., 2018; Kaunzner et al., 2019). However, many more techniques (T1 mapping, T2, T2 mapping, R2, R2', SWI, phase, etc.) were linked more or less successfully with iron. Some authors worked on the relation between transverse relaxation time (R2^*^ and R2') and specimen temperature, developing an iron quantification method based on a temperature coefficient (Birkl et al., 2015, 2017). This seems a very interesting approach to investigating the relationship between MR signal, iron, myelin and temperature, and is a good example of what can be done with *ex vivo* material compared with *in vivo*. This technique now needs to be reproduced by other teams to confirm its effectiveness.

The types of anatomical specimens used differed greatly. Some studies used excisional material from surgery, such as gangliogliomas or cavernomas, while others compared pathological samples from a brain bank with healthy controls, or *fresh* specimens from required autopsies or anatomy laboratories. Anatomical specimens also differed in size, with some teams using the whole brain, many others only a thick coronal slice (often from brain banks) and the remainder using a small anatomical part, such as the substantia nigra, putamen, subthalamic nucleus, or medium temporal gyrus.

Regarding pathologies, AD (11 articles), MS (9 articles), PD (3 articles), progressive supranuclear paralysis (PSP) (3 articles), MSA (2 articles) and amyotrophic lateral sclerosis (2 articles) were the most frequently studied conditions.

Regarding the physical techniques used for iron quantification, ICP-MS was the one most often used (6 articles), with LA-ICP-MS performed in one study (Wisnieff et al., 2015). The second most frequent technique (4 articles) allowing for mapping was XRF. An interesting technique based on the substance's magnetic characteristics, known as superconducting quantum interference device magnetometry (SQUID), was used to further characterize the paramagnetic properties of the particles, allowing, for example, ferrihydrite crystal to be differentiated from magnetite/maghemite (Birkl et al., 2015; Bulk et al., 2018b).

Given that physical techniques require expensive materials, physicians, expert technicians and sound knowledge of physics, histology remains of interest because of its low cost, simplicity and opportunities for mapping. Perls' staining was the most frequently used technique (19 articles for Perls and 8 articles for Perls + DAB), followed by Turnbull + DAB + ammonium sulfide (5 articles).

Table 2 sums up all the different combinations of MRI and histological or physical techniques.

**Table 2:** Summary of all combinations of MRI sequences with histological or physical technique.

**MRI**	**Histological or physical technique**	**References**
T1 or T1 map	Perls	Matsuda et al., 2017, **Lee et al.**, **2018**
T1 PSIR	Perls + DAB	**Wallace et al.**, **2016**
T1 PSIR	Turnbull + DAB	**Wallace et al.**, **2016**
T2 or T2map	Perls	Chen et al., 1989, Fujii et al., 2007, Tatsumi et al., 2008, Matsusue et al., 2008, De Reuck et al., 2011, Massey et al., 2012, Liem et al., 2012, Foroutan et al., 2013, van Veluw et al., 2016, Massey et al., 2017, Matsuda et al., 2017, **Lee et al.**, **2018**
T2 or T2 map	AAS	**Chen et al.**, **1989**
T2 or T2map	Radio-immune assay kit and peroxidase-antiperoxidase method	Chen et al., 1989
T2 or T2 map	IHC: Ferritin	Fujii et al., 2007, Matsusue et al., 2008, Matsusue et al., 2009, Foroutan et al., 2013
T2^*^ or T2^*^ map	Perls	Tatsumi et al., 2008, De Reuck et al., 2011, Liem et al., 2012, Blazejewska et al., 2013, Foroutan et al., 2013, **Lee et al.**, **2016**, van Veluw et al., 2016, De Reuck et al., 2017, Matsuda et al., 2017, **Lee et al.**, **2018**
T2^*^ or T2^*^ map	Perls + DAB	Dhenain et al., 2002, van Rooden et al., 2009, Meadowcroft et al., 2015, **Bulk et al.**, **2018b**
T2^*^ or T2^*^ map	IHC: Ferritin	Foroutan et al., 2013, Dusek et al., 2016
T2^*^ or T2^*^ map	Turnbull + DAB + Ammonium sulfide	Dusek et al., 2016
T2^*^ or T2^*^ map	XRF	Zheng et al., 2012
T2^*^ or T2^*^ map	AAS	Dusek et al., 2016
R1	Perls	House et al., 2008, Stüber et al., 2014
R1	ICP-AES	**House et al.**, **2008**
R1	PIXE	**Stüber et al.**, **2014**
R2	Perls	**House et al.**, **2008****, Walsh et al.**, **2013**
R2	AAS	**House et al.**, **2007**
R2	ICP-AES	**House et al.**, **2008**
R2	ICP-MS	**Langkammer et al.**, **2010****, Langkammer et al.**, **2012**
R2	XRF	**Antharam et al.**, **2012**
R2' and TcR2'	ICP-MS	**Birkl et al.**, **2015**
R2' and TcR2'	SQUID	**Birkl et al.**, **2015**
R2^*^	Perls	**Walsh et al.**, **2013**, Stüber et al., 2014, Wisnieff et al., 2015
R2^*^	Perls + DAB	Fukunaga et al., 2010, Bagnato et al., 2011, Kwan et al., 2012, Meadowcroft et al., 2015, Wallace et al., 2016
R2^*^	Turnbull + DAB	Wallace et al., 2016
R2^*^	Turnbull + DAB + Ammonium sulfide	Bagnato et al., 2011, **Dusek et al.**, **2016**, **Bagnato et al.**, **2018**, Hametner et al., 2018,
R2^*^	Ferritin : IHC	Fukunaga et al., 2010, Bagnato et al., 2011, Kwan et al., 2012, Dusek et al., 2016, **Birkl et al.**, **2017**
R2^*^	Ferrozine assay	**Hametner et al.**, **2018**
R2^*^	ICP-MS and LA-ICP-MS	**Yao et al.**, **2009**, **Langkammer et al.**, **2010****, Langkammer et al.**, **2012****, Wisnieff et al.**, **2015**
R2^*^	PIXE	**Stüber et al.**, **2014**
R2^*^	XRF	**Antharam et al.**, **2012**
R2^*^	AAS	**Dusek et al.**, **2016**
R2^*^	EPR	**Bulk et al.**, **2018a**
R2^*^	SQUID	**Bulk et al.**, **2018a**
TcR2^*^	IHC : Ferritin	**Birkl et al.**, **2017**
SWI	Perls	**Lee et al.**, **2018**
SWI	Perls + DAB	Schrag et al., 2010b
SWI	Turnbull + DAB	Dal-Bianco et al., 2017
SWI	XRF	Hopp et al., 2010, Zheng et al., 2012
Phase	Perls	**Walsh et al.**, **2013**
Phase	Perls + DAB	Bagnato et al., 2011
Phase	Turnbull + DAB + Ammonium sulfide	Bagnato et al., 2011
Phase	IHC: Ferritin	Bagnato et al., 2011
Phase	ICP-MS	**Langkammer et al.**, **2012**
Phase	XRF	**Hopp et al.**, **2010**
QSM	Perls	Stüber et al., 2014, **Sun et al.**, **2015**, Wisnieff et al., 2015, **Lee et al.**, **2018**
QSM	Perls + DAB	Kaunzner et al., 2019
QSM	Turnbull + DAB + Ammonium sulfide	**Wiggermann et al.**, **2017**, **Hametner et al.**, **2018**
QSM	ICP-MS and LA-ICP-MS	**Langkammer**, **2012**, **Tan et al.**, **2014**, **Wisnieff et al.**, **2015**
QSM	XRF	**Zheng et al.**, **2013**
QSM	Ferrozine assay	**Hametner et al.**, **2018**

AAS, atomic absorption spectrometry; CAA, cerebral amyloid angiopathy; DAB, diaminobenzidine; EPR, electron paramagnetic resonance; ICP-AES, inductively coupled plasma atomic emission spectrometry; ICP-MS, inductively coupled plasma mass spectrometry; IHC, immunohistochemistry LA-ICP-MS, laser ablation ICP-MS; MSA, multiple system atrophy; PIXE, particle induced X-ray emission; PSIR, T1 phase sensitive inversion recovery; QSM, quantitative susceptibility mapping; SQUID, superconducting quantum interference device magnetometer; SWI, susceptibility weighted images; TcR2^*^, temperature coefficient R2^*^; XRF, X-ray fluorescence. Quantitative correlations are indicated in bold.

Although correlations between *in vivo* MRI (especially R2^*^ and QSM) and Hallgren's equations yielded remarkably reproducible results across the different teams (Ghassaban et al., 2018), many centers tried to confirm these by correlating MRI and histological/physical data. We selected 25 articles because one of the goals of the studies they described was to quantify iron using a histological/physical technique and correlate it with MRI sequences. All these studies are summarized in Table 3. Relevant MRI sequence acquisition parameters are provided. When available, Pearson (*r*) or Spearman (ρ) correlation coefficients or coefficients of determination (*r*^2^) are given in Column 6 with their associated *p*-value. Linear equation parameters are set out in Column 7. As only relevant or overall results are given, they do not reflect all the correlations found in the studies.

**Table 3:** List of all quantitative studies with corresponding correlation parameters.

**References**	**Iron quantification method/field strength (tesla)**	**Resolution (mm)** **XxYxZ^**a**^/ acquisition time/ number of echoes**	**Fixation period before MRI**	**Histological or physical technique**	**Linear regression arameters *r* = Pearson coefficient correlation *r*^**2**^ = coefficient of determination**	**Linear correlation equation A x [Fe] + B**
Chen et al., 1989	T2 (1.5T)	5 NA NA	0	Iron: AAS	*p* = 0.1	No correlation
House et al., 2007	R2 (4.7T)	0.6 × 0.3 × 4 NA 7	0	Iron: AAS	*r* = 0.797, *p* < 0.0001	A = 0.098 B = 16.2
House et al., 2008	R1 (1.4T) R2 (1.4T)	NA NA NA	0	Iron: ICP-AES	R1: *r* = 0.72, *p* < 0.0001 R2: *r* = 0.70, *p* < 0.0001	R1: A = 0.0034, B = 0.9 R2: A = 0.031, B = 10.0
Yao et al., 2009^b^	R2^*^ (1.5,3,7T)	0.7x0.7x0.7 3h 00 min 00 s 5	NA	Iron: ICP-MS	*r* = 0.93, *p* < 0.05^b^	A = 0.0099^b^B = 1.71
Langkammer et al., 2010	R2 (3T) R2^*^ (3T)	1 × 1 × 4 0h 06 min 27 s 32 1 × 1 × 4 0h 07 min 35 s 12	0	Iron: ICP-MS	R2: *r* = 0.67, *p* < 0.001 R2^*^: *r* = 0.90, *p* < 0.001	R2: A = 0.04, B = 8.5 R2^*^: A = 0.27, B = 14.3
Hopp et al., 2010	Phase (1.5T)	0.5 × 0.5 × 2 NA NA	NA	Iron: XRF	Phase: *r*^2^ = 0.81, *p* < 0.0001	Phase: A = 0.0025, B = −0.13
Antharam et al., 2012	R2 (14.1T) R2^*^ (14.1T)	0.062 × 0.062 × 0.08 1h 40 min 00 s 15 0.062 × 0.062 × 0.08 1 h 30 min 00 s 18	0	Iron: XRF	R2: *r*^2^ = 0.48 R2^*^: *r*^2^ = 0.43	NA NA
Langkammer, 2012	QSM (3T)	0.5 × 0.5 × 2 0 h18 min 00 s NA	0	Iron: ICP-MS	*r* = 0.87, *p* < 0.001	A = 0.00097, B = −0.037
Langkammer et al., 2012	R2^*^ (3T) Phase (3T)	0.5 × 0.5 × 2 0 h 17 min 27 s 2	0	Iron: ICP-MS	R2^*^: *r* = 0.37 *p* < 0.01 Phase: *r* = −0.14, *p* = 0.33	R2^*^: A = 0.12, B = 22.38 Phase: NA
Walsh et al., 2013	T2 (4.7T) R2 (4.7T) R2^*^ (4.7T) Phase (4.7T)	0.25 × 0.25 × 1–2 0h 06 min 48 s NA 0.8–1 × 1 × 4–5 0 h 15 min 36 s 18–24 0.8–1 × 1 × 2	0	Iron: Perls	T2: *r*^2^ = 0.511–0.650 *p* < 0.001 R2: *r*^2^ = 0.489–0.615 *p* < 0.001 R2^*^: *r*^2^ = 0.628–0.685 *p* < 0.001 Phase: *r*^2^ = 0.441–0.596 *p* < 0.001	NA NA NA NA
		0 h 08 min 54 s 10 0.5 × 0.5 × 2 0 h 06 min 36 s NA				
Zheng et al., 2013	QSM (3T)	0.5 × 0.5 × 0.7 NA 11	6 hours	Iron: XRF	*r* = 0.74-0.87 *p* = NA	A = 0.79-0.80,B = −3.66-+10.81
Stüber et al., 2014^c^	R1 (7T) R2^*^ (7T) QSM (7T)	0.2 × 0.2 × 0.2 NA 2 0.2 × 0.2 × 0.2 NA 8 0.2 × 0.2 × 0.2 NA 8	30 days	Iron: PIXE	R1^c^: *r* = 0.871 *p* < 0.001 R2^*^^c^: *r* = 0.933 *p* < 0.001 QSM^c^: *r* = 0.812 *p* < 0.001	A = 0.249^d^, B = 1.129 A = 0.0526^d^, B = 45.87 A = 0.000143^d^, B = −0.0251
Tan et al., 2014	QSM (3T)	NA × NA × 0.4 0 h 02 min 29 s NA	NA	Iron: ICP-MS	*r* = 0.86 *p* < 0.01	NA
Birkl et al., 2015	R2' (3T) TcR2' (3T)	1 × 1 × 2 0 h 20 min 0 s 6 (T2^*^)	0	Iron: ICP-MS	R2': *r*^2^ = 0.94 *p* < 0.001 TcR2': *r*^2^ = 0.98 *p* < 0.001	A = 0.11, B = 8.85 A = −0.004, B = 0.03
Sun et al., 2015	QSM (4.7T)	0.8–1 × 1 × 2 0 h 08 min 54 s 10	0	Iron: Perls	*r*^2^ = 0.62-0.86 *p* < 0.001	NA
Wisnieff et al., 2015	R2^*^ (3T) QSM (3T)	0.7 × 0.7 × 0.7 NA 12	NA	Iron: LA-ICP-MS	NA	NA
Dusek et al., 2016	R2^*^ (7T)	0.5 × 0.5 × 2 0 h 35 min 00 s 8	NA	Iron:Turnbull + DAB + ammonium sulfide and AAS	Turnbull: *r*^2^ = 0.57-0.64 *p* = 0.01-0.02 AAS: *r*^2^ = 0.70, *p* < 0.001	NA
Lee et al., 2016	T2^*^ (7T)	0.136 × 0.136 × 0.5 0 h 25 min 0 s 10	3-5 months	Iron: Perls	*r* = −0.45 -−0.63 *p* < 0.001	A = −8.05-−12.64^e^B = 10.83-11.95^e^
Wallace et al., 2016	PSIR	0.3 × 0.3 × 0.6 6 h 35 min 0 s NA	Several months	Iron: Perls + DAB and Turnbull + DAB	*r* = 0.18 *p* = NA	NA
Birkl et al., 2017	R2^*^ (3T) TcR2^*^ (3T)	0.5 × 0.5 × 2 0 h 06 min 28 s	NA	Ferritin: IHC (light chain)	R2^*^: *r* = 0.14 *p* = 0.36 TcR2^*^: *r* = −0.66 *p* < 0.001	A = 0.06, B = 37.51 A = −0.0032, B = −0.7756
		6				
Wiggermann et al., 2017	QSM (3T)	0.4 × 0.4 × 0.8 0 h 14 min 20 s NA	NA	Iron: Turnbull + ammonium sulfide + DAB	*r*^2^ = 0.02 *p* = 0.35	NA
Bagnato et al., 2018	R2^*^ (7T)	0.7 × 0.7 × 0.7 2 h 16 min 52 s 5	Several years	Iron: Turnbull + DAB+ ammonium sulfide	*r* = 0.42-0.86 *p* < 0.01	NA
Bulk et al., 2018a	R2^*^ (7T)	× 0.1 × 0.1 3 h 30 min 0 s 4	4 weeks – 3 years	Iron: EPR and SQUID	ρ = 0.12^f^ *p*>0.05	NA
Bulk et al., 2018b	T2^*^ (7T)	/ 3 h 30 min 0 s 1	NA	Iron: Perls + DAB	*r* = 0.6 *p* = NA	NA
Lee et al., 2018	T1map (7T) T2map (7T) T2^*^map (7T) T2^*^/T2 (7T) T2^*^/T2^2^ (7T) SWI (7T) QSM (7T)	0.136 × 0.136 × 0.5 NA 10–15	NA	Iron: Perls	T1map: *r* = −0.37**–** −0.11 *p* < 0.001 T2map: *r* = −0.61– −0.35 *p* < 0.001 T2^*^map: *r* = −0.65– −0.11 *p* < 0.001 T2^*^/T2: *r* = −0.64– −0.15 *p* < 0.001 T2^*^/T2^2^: *r* = −0.43– −0.26 *p* < 0.001 SWI: *r* = −0.65– −0.18 *p* < 0.005 QSM: *r* = 0.29–0.44 *p* < 0.001	NA
Hametner et al., 2018	R2^*^ (7T) QSM (7T)	0.43 × 0.43 × 0.65 0 h 13 min10 s 4	0	Iron: Turnbull + ammonium sulfide + DAB Ferrozine assay	Turnbull: R2^*^: *r* = 0.619 *p* < 0.0001 Turnbull: QSM: *r* = 0.445 *p* < 0.0001 Ferrozine: R2^*^: *r* = 0.738 *p* = NA Ferrozine: QSM: *r* = 0.755 *p* = NA	A = 0.51236, B = 15.2827 A = 0.0006723B = −0.04618 A = 1.10, B = −7.13 A = 0.00196B = −0.10

AAS, atomic absorption spectrometry; CAA, cerebral amyloid angiopathy; DAB, diaminobenzidine; EPR, electron paramagnetic resonance; ICP-AES, inductively coupled plasma atomic emission spectrometry; ICP-MS, inductively coupled plasma mass spectrometry; IHC, immunohistochemistry; LA-ICP-MS, laser ablation ICP-MS; MSA, multiple system atrophy; NA, not available; PIXE, particle induced X-ray emission; PSIR, T1 phase sensitive inversion recovery; QSM, quantitative susceptibility mapping; SQUID, superconducting quantum interference device magnetometer; SWI, susceptibility weighted images;TcR2^*^, temperature coefficient R2^*^; XRF, X-ray fluorescence.

a XxY, in-plane resolution; Z, thick section

b iron correlated with R2^*^/tesla rather than R2^*^

c linear equation parameters determined with myelin inference

d correlation with iron expressed in kg/mg dry weight determined by PIXE

e linear equation parameters determined with neuromelanin inference

f *Spearman correlation coefficient*.

Quantitative correlations have been attempted with many physical techniques, but advances in digitization and image post-processing software have recently led to a renewal of interest in Perls' or Turnbull staining. Table 4 lists all the articles describing MRI + Perls' or Turnbull staining. Staining techniques and slice thickness are provided, along with the digitization step and a brief description of the correlation methodology. *Visual* always refers to qualitative assessment.

**Table 4:** Technical description of Perls' and Turnbull staining with brief description of correlation methodology with MRI.

**References**	**Technique**	**DAB**	**Slice thickness**	**Digitization**	**Correlation methodology**
Chen et al., 1989	Not described	No	/	No	Visual
Dhenain et al., 2002	30 min 2% PF 2% HCL	Yes	7 μm	Yes	Visual
Fujii et al., 2007	Not described	No	/	No	Visual
House et al., 2008	1 h 7% PF 3% HCL 37 °C	No	6 μm	Yes	Visual
Tatsumi et al., 2008	Not described	No	/	No	Visual
Matsusue et al., 2008	Not described	No	/	No	Visual
van Rooden et al., 2009	15 h 7% PF 3% HCL	Yes	5 μm	No	Visual
Schrag et al., 2010b	20 min 10% PF 20% HCL	Yes	8 μm	No	Visual
Fukunaga et al., 2010	2 h 5%PF 5% HCL 37 C	Yes	20 μm	No	Visual
Bagnato et al., 2011	15 min 1% PF 1% HCL	Yes	3-7 μm	Yes	Visual
De Reuck et al., 2011	Not described	No	12 μm	No	Visual
Massey et al., 2012	Not described	No	20 μm	Yes	Visual
Kwan et al., 2012	1 h 2% PF 2% HCL	Yes	6 μm	No	Visual
Liem et al., 2012	Not described	No	10 μm	No	Visual
Blazejewska et al., 2013	Not described	No	5 μm	Yes	Visual after coregistration
Foroutan et al., 2013	Time not given 10% PF 20% HCL	No	5 μm	Yes	ROI to ROI
Walsh et al., 2013	30 min 1% PF 1% HCL	No	8mm	Yes	ROI to ROI with optical density
Meadowcroft et al., 2015	30 min 2% PF 2% HCL	Yes	60 μm	Yes	Visual
Stüber et al., 2014	Not described	No	30 μm	No	Visual
Sun et al., 2015	30 min 1% PF 1% HCL	No	8mm	Yes	ROI to ROI with optical density
Wisnieff et al., 2015	1.5 h 5% PF 10% HCL then microwaved	No	10 μm	Yes	Visual after coregistration
Lee et al., 2016	Time not given 20% PF 20% HCL	No	50 μm	Yes	Voxelwise correlation analyses after coregistration
van Veluw et al., 2016	Not described	No	6 μm	No	Visual
Wallace et al., 2016	2H 50% PF 2.5%HCL 37°C	Yes	40 μm	Yes	Mean intensity in ROI after coregistration
De Reuck et al., 2017	Not described	No	/	No	Visual
Massey et al., 2017	Not described	No	20 μm	No	Visual
Matsuda et al., 2017	Not described	No	6 μm	Yes	Visual after coregistration
Bulk et al., 2018b	30 min 1% PF HCL for pH <5.5	Yes	20 μm	Yes	Pixel-based spatial correlation after coregistration
Kaunzner et al., 2019	30 min 4% PF 4% HCL	Yes	5 μm	Yes	Visual
Lee et al., 2018	Time not given 20% PF 20% HCL	No	50 μm	Yes	Voxelwise correlation analyses after coregistration
Bagnato et al., 2011	90 min AS then 15 min 10% Pf 0.5% HCL	Yes	3-7 μm	Yes	Visual
Dusek et al., 2016	90 min AS then 15 min 10% Pf 0.5% HCL	Yes	5 μm	Yes	Area fraction in ROI after coregistration
Wallace et al., 2016	2 h 50% Pf 2.5% HCL 37 °C	Yes	40 μm	Yes	Mean intensity in ROI after coregistration
Wiggermann et al., 2017	90 min AS then 15 min 10% Pf 0.5% HCL	Yes	10 μm	Yes	Area fraction and mean intensity in ROI after coregistration
Bagnato et al., 2018	90 min AS then 15 min 10% Pf 0.5% HCL	Yes	10 μm	Yes	Mean intensity in ROI after coregistration
Hametner et al., 2018	90 min AS then 15 min 10% Pf 0.5% HCL	Yes	10 μm	Yes	Mean intensity in ROI without coregistration

*AS, Ammonium sulfide, HCL, hydrochloric acid; PF, potassium ferrocyanide; Pf, potassium ferricyanide; ROI, region of interest. when reaction temperature not given, assume room temperature. Solid line separates Perls' procedures (above) from Turnbull procedures (below)*.

## Discussion

### Qualitative Histological Studies and MRI

Since the advent of MRI, constant efforts have been made to improve understanding of what the images show. Iron is a perfect candidate in this respect, as its paramagnetic property means that it is observable by MRI. Furthermore, iron may play a role in many neurological disorders. With the aim of better understanding what we can see in *in vivo* MRI and being certain that it is iron, it is obviously useful to look for correlations between MRI sequences and iron histology.

In the case of AD, Dhenain et al. (2002) and House et al. (2008) failed to correlate iron in senile plaques with T2^*^ hypointensities or R1 and R2, in contrast to van Rooden et al. (2009), who concluded that amyloid plaques and cerebral amyloid angiopathy (CAA) are collocated with iron. Meadowcroft et al. (2015) confirmed the role of iron in the transverse relaxation rate in Aβ plaques, but when they compared deferoxamine iron-chelated tissue with unchelated tissue using Perls + DAB, they demonstrated that iron is not the sole determinant of the relaxation rate, and the fibrillar nature of Aβ plaques also has a major role in transverse relaxation. Bulk et al. (2018a) demonstrated a good spatial correlation between iron revealed by Perls + DAB and T2^*^ in the whole cortex of patients with AD, and not only in Aβ plaques as previously described. They compared early-onset AD with late-onset, and showed that iron-rich microglial cell recruitment is responsible for a higher iron concentration in the early-onset stage than in controls or the late-onset stage. They confirmed that iron is not the sole determinant of T2^*^-weighted contrast, and that myelin also plays a decisive role, with a good spatial correlation between iron and myelin as already described elsewhere (Fukunaga et al., 2010; Wallace et al., 2016). In another article, Bulk et al. (2018b) described significantly higher values of R2^*^, a high concentration of ferrihydrite, and a higher magnetic moment of magnetite/maghemite particles in the middle temporal gyrus of patients with AD.

Schrag et al. (2010b) found a correlation between SWI and hemosiderin in brain microbleeds in patients with CAA and AD that was subsequently confirmed by De Reuck et al. (2011) and van Veluw et al. (2016), with correlations between T2/T2^*^and Perls. Interestingly, some acute hemorrhages were not stained, unlike old hemorrhages, confirming the absence of heme-iron staining by Perls and the gradual breakdown of blood products into hemosiderin.

For PD, Blazejewska et al. (2013) described an overlapping of Perls' staining with T2^*^ hypointensity after image coregistration in the substantia nigra of patients with PD and controls. Regions with high neuromelanin did not correlate with Perls' staining and T2^*^ hypointensities, confirming that iron stored in neuromelanin cannot be stained by Perls, probably owing to its strong affinity with neuromelanin proteins (Zucca et al., 2017). The authors found that nigrosome-1 had a low iron concentration, which was confirmed by Massey et al. (2017), with no differences between patients with PD and controls, compared with patients with PSP, in whom nigrosome-1 appears hyperintense in Perls' staining. In patients with PSP, relative to controls, Foroutan et al. (2013) also described an increase in iron in the substantia nigra and globus pallidus highlighted both by T2^*^ and by Perls. In MSA, (Matsusue et al., 2008, 2009) revealed ferritin deposits in the dentate nucleus that were associated with T2^*^ weighted image hypointensity. In the putamen, hypointensities on T2^*^ were also correlated with ferritin and extensive iron staining.

From a purely anatomical point of view, besides the low concentration of iron in nigrosome-1 evoked earlier, more intense staining was reported in the anteromedial part of the substantia nigra pars reticulata where pallido-striato-nigral fibers begin (Blazejewska et al., 2013; Lee et al., 2016, 2018; Massey et al., 2017). This was confirmed by another study using LA-ICP-MS (Matusch et al., 2011). In the subthalamic nucleus, iron-rich regions were found to be located in the anteromedial part of the nucleus, with iron levels gradually decreasing toward the posterolateral part. This was assessed both by MRI-Perls analysis (Massey et al., 2012) and by other techniques (Dormont et al., 2004; de Hollander et al., 2014).

For Wilson's disease, Dusek et al. (2016) found that iron and copper levels were higher in the putamen of patients than controls, but only iron concentration was correlated with R2^*^.

In MS, Bagnato et al. (2011) compared R2^*^ and phase sequences at 7T with Perls + DAB staining and several antibodies for immunohistochemistry (ferritin, oligodendrocytes, microglia…). They found that both oligodendrocytes and microglia were iron-rich in the normal-appearing white matter of patients with MS, compared with oligodendrocytes only in controls. Ferritin- and iron-enriched activated microglia/macrophages were also found surrounding the core of the lesion, but not in the core itself, except in the perivascular space where extracellular hemosiderin can be found. When Walsh et al. (2013) explored 30 MS lesions from three different patients, they found heterogeneous patterns of Perls' staining and R2^*^ and phase signal in these lesions. Wisnieff et al. (2015) concluded that iron is responsible for positive susceptibility values (QSM) in demyelinated lesions, but in normal-appearing white matter, the myelin contribution prevents the analysis of iron by QSM. The ferritin count also differs between the core and the edge of the lesions, with more ferritin in the edge, as revealed by Birkl et al. (2017). According to Wiggermann et al. (2017), few MS lesions contain iron diffusively, and QSM should not be interpreted in terms of the lesions' bulk magnetic susceptibility, but relative to abnormalities in the surrounding normal-appearing white matter. Bagnato et al. (2018) confirmed that both iron and myelin contribute to the R2^*^ signal, but in different ways, depending on which brain structure is evaluated. They stated that iron is a stronger contributor to R2^*^ changes than myelin. We are of the view that it is fiber orientation more than myelin quantity that plays a role in R2^*^ variations. For an extended review of MS and iron, see Stüber et al. (2016), in which a diagram based on lesion chronology tries to explain the various patterns found both in iron histology and in iron-MRI sequences. These authors also point out that the basal ganglia appear richer in iron in patients with MS than in controls.

We can see that MRI/histology visual comparisons can be a mine of information and provide a set of data that are helpful for clinicians and neuroscientists and which cannot be obtained with other techniques. However, visual comparisons are often more subjective than strict quantitative correlations.

### Quantitative Histological Studies With Physical Techniques and MRI

Physical techniques are regarded as the benchmark for detecting trace elements such as iron. House et al. (2008), House et al. (2007) were the first to find a correlation between relaxation parameters (R1, R2) and iron concentration, as determined by AAS or by inductively coupled plasma atomic emission spectrometry (ICP-AES) [*r* = 0.72 (R1) and 0.70-0.797 (R2)]. Results for R2 are remarkably consistent across studies, regardless of which technique is used: *r* = 0.67 with ICP-MS (Langkammer et al., 2010); *r* = 0.69 with XRF (Antharam et al., 2012). Linear correlation equation parameters are of the same order of magnitude. For R2^*^, *r* ranges from 0.37 to 0.93, with a significant *p*-value, in line with *in vivo* studies (Ghassaban et al., 2018). The degree of correlation depends on which anatomical structure is studied and the number and exhaustiveness of the sampling points. For example, when Langkammer (2012) studied the frontal and occipital gray/white matter interface, conducting correlation analyses of both gray and white matter with iron determined by ICP-MS, they found significant correlations both overall (*r* = 0.37, *p* < 0.01) and for gray matter only (*r* = 0.42, *p* < 0.05), but not for white matter only. They postulated that R2^*^ is affected both by paramagnetic iron and by diamagnetic myelin. By contrast, when they pooled the basal ganglia with white matter, the Pearson correlation coefficient rose to *r* = 0.90 (*p* < 0.001), owing to the richness in iron and relative poorness in myelin of the basal ganglia (Langkammer et al., 2010). To overcome the myelin contribution, Stüber et al. (2014) separated the myelin fraction and iron concentration determined by particle-induced X-ray emission in the paracentral lobule and occipital lobe. A univariate analysis with iron yielded a correlation of *r* = 0.56, but a multivariate analysis with myelin fraction and iron concentration yielded a correlation of *r* = 0.93. This clearly demonstrated that iron and myelin are the two main contributors to R2^*^ in white matter. The authors claimed that fiber orientation contributes to the R2^*^ relaxation rate compared with the R1 rate, which is affected by myelin quantity but not orientation. When Yao et al. (2009) plotted R2^*^ values as a function of field strength, they demonstrated that R2^*^ augments with field strength, and R2^*^ rate of change with field strength is a function of iron concentration (*r* = 0.93). They concluded that R2^*^ depends linearly on iron concentration, at all field strengths studied (1.5–3-7 T). Bulk et al. (2018b) failed to find a correlation between iron (Fe^3+^) measured by electron paramagnetic resonance (EPR) or ferrihydrite and magnetite/maghemite measured by SQUID and R2^*^ in the temporal cortex. This can be explained by the choice of anatomical specimen and by various technical considerations identified by the authors.

Hopp et al. (2010) found a strong correlation between phase images and XRF (*r* = 0.90, *p* < 0.0001), whereas Langkammer (2012) failed to do so. The main difference between these two studies was the exhaustiveness of the sampling. Hopp et al. used several parts of the brain, including the basal ganglia, whereas Langkammer et al. only used the frontal and occipital cortices with their adjacent white matter (owing to their research question). Using QSM, Langkammer et al. (2012) sampled basal ganglia and lobar white matter offering a wide range of iron levels, as measured by ICP-MS. The resulting correlation appeared strong (*r* = 0.87, *p* < 0.001). Zheng et al. (2013) used pixel-by-pixel correlation allowed by XRF mapping to confirm this correlation (*r* = 0.74 for right side and *r* = 0.87 for left side). However, they only considered basal ganglia (putamen, caudate nucleus, and globus pallidus) from one individual in the analysis. Other studies have reported correlations ranging from *r* = 0.76 to *r* = 0.86 (Stüber et al., 2014; Tan et al., 2014; Hametner et al., 2018), in accordance with *in vivo* studies (Ghassaban et al., 2018). The QSM method these authors used included homogeneity-enabled incremental dipole inversion (Langkammer et al., 2012; Hametner et al., 2018), threshold k-space division (TKD) (Zheng et al., 2013), morphology enabled dipole inversion (MEDI) (Tan et al., 2014; Wisnieff et al., 2015), and calculation of susceptibility through multiple orientation sampling (COSMOS) (Wisnieff et al., 2015).

*Ex vivo* quantitative studies performed in different brain subregions and with different physical techniques confirm *in vivo* correlations made with Hallgren and Sourander's equations, especially for R2^*^ and QSM. In the basal ganglia and cortex, iron accounts for most of the recorded signal, compared with white matter in which myelin quantity and anisotropy form part of the signal.

Mapping is difficult to perform with physical techniques, and has so far only been undertaken by a few research teams. Technical progress in image digitization now makes it possible to perform MR signal correlations directly with colorimetry.

### Quantitative Histological Studies With Staining and MRI

We have seen how a robust protocol is essential for quantitative studies, but it is truer still for studies with staining. Colorimetry can be described as a semiquantitative tool, as it can only provide an indirect estimation based on optical density or pixel intensity, not a direct concentration of an element (iron). However, the same could be said of all available MRI techniques that only quantify a signal (relaxation rate, susceptibility), rather than directly measuring iron concentrations. Despite these considerations, some authors, using a good image coregistration methodology, have successfully demonstrated the utility of histology/MRI correlation that does not solely rely on simple visual assessment.

Walsh et al. (2013) compared R2, R2^*^, T2 and phase imaging with Perls' staining on 8-mm slices through the whole brain. Each slice was photographed before and after staining. For each set of slice photographs, grayscale conversion was performed, adjusting the window and level of the stained photographs to match those of the unstained photographs using two reference points: background and a region of unstained white matter. The differences in intensity between the stained and unstained photographs were then normalized after division by the difference in intensity between the background and the unstained white-matter reference region for each slice. An image of relative optical density was thus produced, where a higher value corresponded to greater iron staining. Several regions of interest (ROIs) in the basal ganglia were used for analysis. This technique was first described by Bizzi et al. (1990) in primates. These authors reported a range of values for *r*: 0.71-0.81 for T2 fast spin-echo, 0.70-0.78 for R2, 0.79-0.83 for R2^*^, and 0.66-0.77] for phase images. All were significant (*p* < 0.001). Using exactly the same protocol for QSM (total variation regularization approach), Sun et al. (2015) found a range of 0.79-0.93 (*p* < 0.001).

Dusek et al. (2016) used a different histological technique. After digitization and color deconvolution of DAB-specific slices with ImageJ (Schindelin et al., 2012), 8-bit grayscale images were inverted and thresholded so that only gray values above 234/255 (empirically determined), representing the most intense staining, were segmented. An index representing the proportion of tissue with a positive Turnbull signal was defined as the area fraction reflecting the percentage of pixels with an intensity above 234. Values were averaged for basal ganglia ROIs. The authors found *r* values of 0.75-0.8 (*p* = 0.01-0.02) with R2^*^, consistent with the AAS technique (*r* = 0.84) applied to the same sample. Wiggermann et al. (2017) used roughly the same methodology to determine area fraction, and they also determined the integrated density of iron, based on the 8-bit gray values. Although they failed to find a correlation with QSM, they focused on MS lesions, and as has already been said, this is greatly complicated by low sampling exhaustiveness and the complex relation between MS lesions, myelin and iron. When Bagnato et al. (2018) used approximately the same histological quantification method and the same iron staining in MS as Wiggerman et al, but with ROIs in different locations across the brain, they found *r* values of 0.42-0.86, with R2^*^ depending on the selected region (cortex or diffuse white-matter injury vs. basal ganglia).

After digitization and binarization of Perls' images of the substantia nigra, Lee et al. (2016, 2018) coregistered histology/MRI images by downsampling histology images and upsampling MRI images, and then carried out a rigid transformation for a perfect match, in order to perform voxel-by-voxel correlations (considering the histological images as 3D structures). These authors found that the T2^*^ map was more closely correlated with iron (*r* = −0.65 **-** −0.11) than the T1map, T2map, or T2^*^/T2 and T2^*^/T2^2^, which appeared to correlate better with neuromelanin. Surprisingly, results for SWI were in the same order of magnitude as those for the T2^*^map, but QSM (MEDI) results appeared less closely correlated with iron than SWI.

After digitization, Bulk et al. (2018a) eliminated background noise before downsizing histological images to MRI resolution to facilitate registration. Images were then coregistered by non-linear transformation. ROI masks were applied and pixel-based spatial correlation of the signal intensities was undertaken. The authors found a correlation (*r* = 0.6, *p* not available) between Perls + DAB and T2^*^ in the frontal gyrus sample of controls.

After digitizing the images, Hametner et al. (2018) carried out color deconvolution before converting the images into 8-bit grayscale and inverting them. The histological and MR images were not coregistered. Mean intensities were measured in ROIs located in exactly the same regions on both sets of images. The authors found correlations of *r* = 0.62 for R2^*^ and *r* = 0.45 for QSM (*p* < 0.0001). These results were poorer than those yielded by a ferrozine assay on the same specimens (*r* = 0.74 for R2^*^ and *r* = 0.76 for QSM). They also plotted the iron concentration from the ferrozine assay as a function of the iron intensities from staining and found a quadratic correlation. In other words, the more intense the iron concentration, the less sensitive the staining. This could explain, at least in part, the different results for each method.

Although quantitative studies with staining are less precise than those using physical techniques, they still offer a reliable estimation of iron quantities. In addition to mapping and the possibility of visually assessing stain slices at cellular resolution, iron staining allows users to selectively stain non-heme Fe^3+^, Fe^2+^, or both. By contrast, most physical techniques quantify all the different iron forms (heme iron and non-heme Fe^3+^ and Fe^2+^) without distinction. However, for a good correlation, several key points have to be borne in mind, as each of these techniques can lead to biases.

### Limitations of *ex vivo* MRI, Histological Techniques and Image Coregistration

#### Ex Vivo MRI

**Figure 3:**
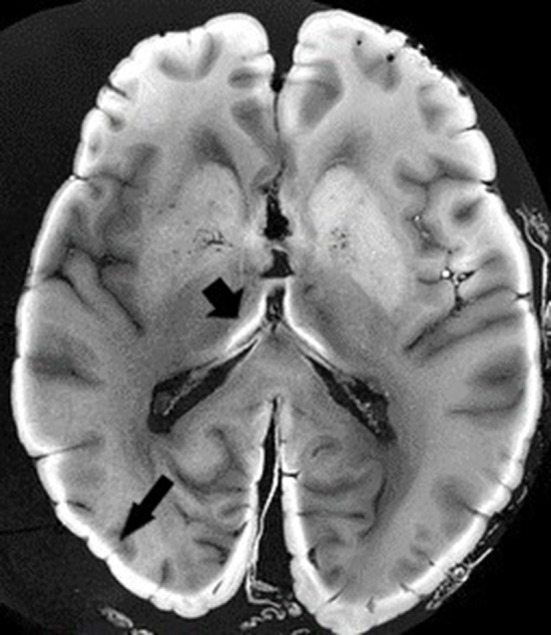
*Ex vivo* MRI. T1-weighted sequence. Black arrows depict light band of formalin-fixed tissue. Image obtained during experimental procedures with all appropriate ethical approval. Included for illustrative purposes.

*Ex vivo* MRI shares many sources of artifacts with *in vivo* MRI, but also has specific ones that it is important to be aware of before beginning any scans. First, there is the delay between the death and the MR scan, if the whole head is imaged before brain extraction, or between the death and the brain extraction. Autolysis begins as soon as death occurs, with an increase in extracellular water content, a pH reduction, and a reduction in temperature, leading to shorter T1 values and longer T2 ones (Dawe et al., 2009; Tashiro et al., 2015). The goal of chemical fixation is to preserve postmortem tissue in a state close to the living condition. There are many fixative agents around, but the most widely used is formaldehyde. The nature of the fixative solution affects relaxation times and diffusion properties (Shepherd et al., 2009; Birkl et al., 2018). Formaldehyde solutions can be prepared with very different concentrations, even if the most frequently used is 4%, leading to 10% formalin (formaldehyde and formalin are different). This can modify the time needed for the optimum fixation of the brain tissue (Birkl et al., 2018). The volume of material to be fixed is, of course, also important. Fixation reduces T1 relaxation, T2/T2^*^ relaxation and proton density, and increases myelin water fraction (Yong-Hing et al., 2005; Dawe et al., 2009; Birkl et al., 2016; Shatil et al., 2018). The length of immersion in formalin is of crucial interest, as relaxation properties are not modified in the same manner if the brain specimen is fixed for a few days or for several years. This is due to the two-step fixation mechanism. The fixation process initially involves chemical reactions between formaldehyde and water creating methylene glycol, which rapidly penetrates brain tissue, owing to a low molecular weight, creating dehydration. This step is partly reversible by rehydration of the anatomical specimen before imaging (Shepherd et al., 2009). The second step of the fixation process leads to irreversible tissue damage by creating methylene bridges between macromolecules (Birkl et al., 2016). Some authors consider 5–6 weeks in 20% formalin to be the optimum length of time for complete fixation, but this period is very variable, depending on the fixative agent, concentration, and volume of the specimen (Yong-Hing et al., 2005). A long fixation period (e.g., in a brain bank) leads to histological artifacts (van Duijn et al., 2011). In T1-weighted (but not T2- or proton density-weighted) images, the formalin-fixed tissue appears as a light band that gradually widens over time (Figure 3). Dawe et al. (2009) analyzed differences in T2 relaxation caused by formalin fixation, comparing surface and deep brain tissue. T2 values for surface tissue rapidly decreased then plateaued, whereas for deep tissue, they rapidly decreased then slowly rose to a plateau, owing to the ongoing autolysis process. We can speculate that the first step of dehydration was responsible for the rapid decrease, and the second step of protein crosslinking was responsible for the plateauing. However, others argue that water tissue loss plays only a minor role (Birkl et al., 2016). In a recent study, QSM was carried out before death, then at weekly intervals after death for 6 weeks. The brains were in a formalin solution. Postmortem QSM correlated very closely with premortem QSM, and susceptibility remained unchanged throughout the 6 weeks (Evia et al., 2017).

Unlike *in vivo* MRI, the temperature at which the scan is performed affects the signal. T1 and, to a lesser extent, T2 and T2^*^ relaxation times decrease when the temperature drops (Birkl et al., 2016). However, as seen above, we can take advantage of this property and use the temperature coefficient as a measure of paramagnetic susceptibility (Birkl et al., 2015, 2017).

Concerning rehydration periods before MRI, our literature review showed that some authors only immersed the specimen in saline solution just before scan, whereas others soaked the specimen for 12, 24 h or more, changing the solution several times (Shepherd et al., 2009; De Reuck et al., 2011; Blazejewska et al., 2013; Wallace et al., 2016).

The medium in which the brain is imaged is also of particular importance, as it can affect the signal and disturb susceptibility values. Proton-free fluorinated fluids are regarded as the gold standard, as they are inert, produce no MRI signal, and have a similar magnetic susceptibility to tissue (Miller et al., 2011; Shatil et al., 2016). However, availability, cost, and possible interferences with subsequent histology limit their use. Some authors use a solid (paraffin/plastic polymer mixture) or semisolid (agarose, gelatin) medium, to avoid vibrations, but very little is known about their contribution to the MR signal. The present review revealed the use of many different protocols, some involving fluorinated fluids and others directly a formalin or saline solution, while many teams used a solid or semisolid medium.

**Figure 4:**
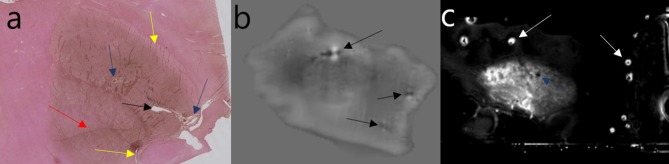
Example of artifacts. Perls + DAB: yellow arrow = tears; black arrow = vascular dilation; blue arrow = paravascular dilation; red arrow = knife mark **(a)**. Phase image: black arrow = vascular blood clot **(b)**. R2* image: white arrows = trapped air bubbles; blue arrow = paravascular dilation **(c)**. Image obtained during experimental procedures with all appropriate ethical approval. Included for illustrative purposes.

Four important sources of artifacts with *ex vivo* MRI are air bubbles, intravascular blood clots, paravascular space dilation, and vibrations in the scanner. The air/tissue interface induces susceptibility artifacts that can seriously compromise the interpretation of susceptibility-based sequences. A liquid medium allows air bubbles to be eliminated by complete immersion and gentle shaking of the container. However, if the anatomical specimen is not correctly embedded (e.g., with gauze), it is exposed to vibrations, yielding uninterpretable images. A semisolid or solid medium often allows vibrations to be avoided, but managing air bubbles is much more complicated and requires considerable experience (Shatil et al., 2016). Another solution is to image the whole head, as many teams did with fresh specimens, but to our knowledge, nobody has yet tried to image a fixed whole head in order to examine iron. Intravascular blood clots can be limited by washing the vessels in the forensic or anatomy laboratory. When the specimen is stored for a long time before imaging, blood seems to dilute in the fixative solution. Paravascular enlargement can be seen *in vivo* (Virchow-Robin space) (Bacyinski et al., 2017), and when the brain is fixed before imaging, the shrinkage seems to accentuate this (Figure 4). It can also be seen in histological studies.

Specimen orientation in the scanner is a theoretical problem for *ex vivo* MRI compared with *in vivo* MRI, especially with small anatomical specimens. It is important to think about this before the scan, in order to avoid having to reorient the histological sections during image post-processing, which is a potential source of image information loss. Furthermore, with small specimens, it can be hard to find an acquisition plane that is commonly used for *in vivo* MRI, making it more difficult to extrapolate from the results. Once again, a rigorous methodology is essential.

In contrast to *in vivo* MRI, whole brains or small parts of the brain can be imaged using a wide variety of radiofrequency coils. Dedicated homebuilt coils can be designed, or dedicated coils for other anatomical body parts (e.g., wrist) can be diverted, in order to achieve a better signal/noise ratio and a better contrast. However, this can impinge on the comparability of results across centers.

The present review showed that scanner protocols for iron-based sequences can differ greatly across studies. The main differences we found lay in the total scan duration, which could range from 2 min to more than 6 h for quantitative studies with a high variability (Table 3), and be very much longer still for other studies. Spatial resolution and slice thickness mainly depended on the scanner's capacities, but it is interesting to note how they varied across studies. The number of signals averaged also differed across studies, as did the number of echoes performed during a multi-gradient echo sequence for T2^*^ and R2^*^. Not only the number of echoes, but also the interecho time and the range of this echo time, are important to precisely describe R2^*^(Péran et al., 2007). QSM can be calculated with single-echo gradient acquisition, but multi-echo gradient acquisition ensures a better signal/noise ratio (Deistung et al., 2016). With QSM, the multitude of post-processing algorithms available can lead to slightly different results (Langkammer et al., 2017). Once again, we need to be cautious when comparing different studies with different QSM or R2/R2^*^ protocols (especially if the histological protocol also differs) (see Table 5).

**Table 5:** Key points to consider in histology, *ex vivo* MRI, iron-based sequences and image coregistration, in order to limit methodological problems and to compare studies.

***Ex vivo* MRI**	**Iron-based sequences**	**Histology**	**Image coregistration**
Time between death and MRI (fresh)	Spatial resolution	Slice thickness	Image file formats
Time between death and fixation (fixed)	Slice thickness	Staining protocol	Up- or downsampling image resolution
Fixation period before MRI	Acquisition time	Type of staining	Non-linear transformation?
Fixative agent	NSA	Fixation time between MRI and histology	Stack procedure?
Degree of putrefaction	Number of echoes (relaxometry)	Tears	Color deconvolution?
Imaging solution	Echo time (relaxometry)	Stretching	Thresholded out background
Rehydration or not (fixed)	Post-processing algorithms (QSM)	Shrinkage	Grayscale conversion
Rehydration time (fixed)		Distortions	
Room temperature		Swelling	
Air/tissue interface (bubbles)		Blood vessel or paravascular space dilation	
Contention technique to avoid vibrations		Knife mark	
Specimen orientation		Sampling	
RF coil type			
Intravascular blood clot or paravascular space dilation			

*NSA, number of signals averaged; QSM, quantitative susceptibility mapping; RF, radiofrequency; ROI, region of interest*.

#### Histological Techniques

MRI has its fair share of random variables, but histology is also a source of limitations that need to be borne in mind. As we can see in Table 4, slice thickness varied greatly across studies, ranging from 5 μm to 8 mm. It is not important when a simple visual assessment is carried out, but the determination of iron intensities may vary according to the thickness of the stain slice. Most of the time, histological slices are several micrometers thick, whereas MRI slices are hundred micrometers for the best resolution, raising questions about the comparability of results. At the micrometer scale, not all the slices can be stained, and sampling is mandatory (e.g., 1 in every 20 slices stained for example), reducing the representativeness of the data. Conversely, some authors used a thickness of 8 mm, with an MRI slice thickness of 2 mm (Walsh et al., 2013; Sun et al., 2015). The intensity of the stained slices may not reflect the MRI signal intensity measure. Along with slice thickness, stain protocols differ greatly across studies, even if we only consider a single staining technique such as Perls' (see Table 4). It should, however, be noted that the Turnbull + DAB procedure after ammonium sulfide seemed to be the standard in many recent studies. The choice of staining procedure (Perls', Turnbull, ferritin immunohistochemistry) and the application (or not) of DAB can make a considerable difference (van Duijn et al., 2013).

Time between MRI and the staining procedure is important, as iron leakage in a formalin solution has been shown to increase over time, leading to correlation difficulties (Gellein et al., 2008; Schrag et al., 2010a; Krebs et al., 2014). However, significant differences only appear for very long-term storage, and are almost negligible for the first 12 weeks.

The last important point concerns histological processing artifacts. Fixation, inclusion, cutting and staining can lead to distortions, tears, shrinkage, swelling, stretching, blade marks, and so on that can affect the correlation process (Figure 4). To avoid these technical limitations, some authors recommend using thick histological sections (Alho et al., 2017). This is an interesting method, for if the main goal of these authors is to perform perfect matching without a quantitative analysis, then homogenizing the slice thicknesses for MRI and histology can presumably also ameliorate quantitative correlations (see Table 5).

#### Image Coregistration

Technological advances now allow histological images to be digitized, and thus to be matched with MR images. A great many processes are needed, involving downsampling, linear and nonlinear transformations, in order to perfectly match the two sets of images. Another possible method is to stack the histological images to create a 3D volume that can be matched with 3D-MRI volume. These procedures are often used by creating an MRI-histology-based atlas (Yelnik et al., 2007; Choe et al., 2011; Adler et al., 2014), but all this image post-processing raises questions about a possible loss of information between the raw data and the final image, even if images are stored in a format with no data compression. We can ask the same questions about the color deconvolution algorithms and grayscale conversion used by some authors in the present review. Thresholding the background in order to keep only certain information is often empirical, according to some authors (Dusek et al., 2016), and can vary from one team to another (see Table 5).

#### Is There an Ideal Coregistration Method?

Given the published literature, we think the simpler the method is, the better it is for final coregistration. The first step is to image your brain sample with minimal artifacts. Unfortunately, there is no perfect method and the best is to experiment for oneself, learning from errors and successes. The second step is to clearly define what is the goal of your histology. Is it to match perfectly the two sets of images or to perform quantitative measures? Often it is necessary to find a good compromise. During histological process, it is important to never forget the final coregistration process in order to limit in the last step (coregistration) as much as possible the need for image manipulation (color deconvolution, downsizing, image transformation…). We would like to give you an ideal standardized method but due to all method possibilities listed above, there is not a perfect way to do and each team have to experiment by oneself with its own available resources.

## Conclusion

We reviewed all the relevant literature concerning *ex vivo* MRI and iron histology and came to three main findings. First, matching *ex vivo* MRI with histology allows for a better understanding of the physiopathology of some neurological disorders in which iron is suspected to play a role, as well as of the radiological anatomy of specific neurological structures. Second, correlating iron-based MRI sequences with iron quantification performed with a reference physical technique shows that MRI sequences can be reliably used for the *in vivo* estimation of iron in the brain. It is interesting to note that results from *ex vivo* MRI were in accordance with *in vivo* data correlated using Hallgren and Sourander's equations. Third and last, data from *ex vivo* MRI correlated with quantitative staining seem to be a good approximation of the results yielded by physical techniques, and histology has the added advantages of being relatively inexpensive and offering opportunities for mapping. However, matching *ex vivo* MRI and histology raises some methodological issues that need to be taken into account before extrapolating to *in vivo* MRI.

## Data Availability

No datasets were generated or analyzed for this study.

## Author Contributions

AD, GA, and PP contributed conception and design of the review. AD and JC wrote the first draft of the manuscript. GA wrote sections of the manuscript. AD, GA, and JC collected data. PC reviewed specifically histological and anatomical parts. PP reviewed specifically MRI parts. All authors contributed to manuscript revision, read and approved the submitted version.

### Conflict of Interest Statement

The authors declare that the research was conducted in the absence of any commercial or financial relationships that could be construed as a potential conflict of interest.
